# Objective and Subjective Physical Activity Assessment in Adolescents with Motor Difficulties

**DOI:** 10.3390/children12040488

**Published:** 2025-04-10

**Authors:** Ermioni Katartzi, Maria Kontou, Ioannis Pappas, Ioannis Trigonis, Thomas Kourtessis

**Affiliations:** 1 Department of Physical Education and Sport Science at Serres, Aristotle University of Thessaloniki, 62100 Serres, Greece; marikont@phed-sr.auth.gr (M.K.); ioanpapp@phed-sr.auth.gr (I.P.); 2 Department of Physical Education & Sport Science, Democritus University of Thrace, 69100 Komotini, Greece; itrigon@phyed.duth.gr; 3 School of Social Sciences, Department of Early Childhood Education & Care, International Hellenic University, 57400 Thessaloniki, Greece; thkourte@ihu.gr

**Keywords:** self-reported physical activity, pedometers, step count, developmental coordination disorder

## Abstract

**Background/Objectives:** Motor difficulties due to developmental coordination disorder seem to exist in adolescence, affecting participation in physical activity. The purposes of the present study were, firstly, to identify motor difficulties and, secondly, to compare objective and subjective weekly physical activity between adolescents with and without motor difficulties. **Methods:** Sixty-nine adolescents who attended secondary school, were screened for motor difficulties, using the Movement Assessment Battery for Children—Second Edition. Then, a motor difficulties group (n = 8) and a group of peers without motor difficulties (n = 7) were formed. Objective physical activity was assessed using Yamax Power Walker EX-510 pedometers (Yamax, Kumamoto, Japan) in a single school week throughout the day, while subjective weekly self-reported physical activity was assessed by the “Godin–Shephard” Leisure-Time Physical Activity Questionnaire in both groups. **Results:** Approximately 7.2% of adolescents denoted significant motor difficulty, and 5.8% suggested that adolescents were “at risk” of having a motor difficulty and monitoring was required. No statistically significant differences were found in objective weekly physical activity between the groups. However, the daily number of steps recorded in both groups was found to be lower than those suggested by literature for health benefits. Moreover, significant differences were shown in the subjective self-reported weekly physical activity regarding participation in high intensity and total weekly physical activity. Adolescents with motor difficulties indicated lower scores, although the majority of both groups could be characterized as active, with substantial benefits for their health. **Conclusions:** Although there is evidence that motor difficulties are prevalent in adolescence, physical activity participation seemed not to have been affected; however, subjective and objective assessment indicated different results. Thus, it is suggested a combination of both methods in assessing physical activity in adolescents with and without motor difficulties in order to provide more robust results.

## 1. Introduction

Developmental coordination disorder (DCD) is a common neurodevelopmental disorder affecting approximately 5–6% of school-aged children, who exhibit fine and/or gross motor skills below the expected level for their age and learning opportunities [1,2,3]. It is estimated that 50–70% of children with DCD continue to be affected in adolescence and even into adulthood by motor difficulties associated with DCD, which appear to persist, causing secondary problems, such as reduced participation in physical activity, team games and sports, poor physical fitness, and obesity [1,2]. The most frequently suggested percentage of DCD prevalence ranges from 5% to 6%, although there is a range from 2% to 20%, in children [1,3]. Additionally, a recent systematic review and meta-analysis about the prevalence of motor difficulties in public primary and secondary schools from different cultures are also ambiguous, as the prevalence of motor difficulties appeared to be from slightly higher [1,2,4] to much lower than previous estimations in school-aged children in South India (0.8%) [5]. It is noted that epidemiological data are largely dependent on how strictly the selection criteria are applied [2].

Regarding prevalence between genders, it was stated that more boys than girls, in a range from 2:1 to 7:1, are affected by DCD [1,6]. However, only the study in South India reported a higher prevalence in girls, with a boy-to-girl ratio of 1:2, in school-aged children (6–15 years old) [5]. This suggests that gender differences in DCD prevalence might vary based on study methods, diagnostic criteria, or cultural factors [2]. Moreover, gender differences in DCD prevalence may be due to underlying neurological system disparities between boys and girls [7]. A comprehensive assessment using standardized tools and clinical assessments is fundamental for the detection of DCD. Assessment tools should also consider factors such as age, culture, and environment in order to design better specific and personalized interventions [8]. The Movement Assessment Battery for Children (Movement ABC-2) [9], in combination with diagnostic criteria from DSM-5, ensures a detailed and accurate identification of DCD, facilitating timely and effective interventions, as shown in studies involving interventions in adolescents [6,10].

Physical activity (PA) is any bodily movement produced by skeletal muscles that results in energy expenditure [11], while leisure-time physical activity (LTPA) is widely recognized as a key domain of physical activity for public health research and intervention [12,13]. It is suggested that children and adolescents (aged 5–17 years) should participate in moderate to vigorous intensity physical activity (MVPA), for a minimum of 60 min daily at least 3 days per week, and also in activities that strengthen muscle and bones. However, participation in more than 60 min of physical activity daily provides further health benefits [14,15]. Regarding the monitoring of physical activity participation using step recording, adolescents who achieve between 10,000 and 11,700 steps daily typically reach 60 min of MVPA [16], although a minimum value of 10,000 steps per day [17] could account for optimal health. Specifically, boys should strive for a minimum of 11,000 steps daily, while girls should aim for at least 10,500 steps. By consistently meeting or exceeding these step goals, adolescents can enhance their overall well-being and long-term health. Evidence from the World Health Organization Regional Office for Europe (WHO/Europe) supported that among adolescents aged 13, only 15% meet the recommended 60 min of daily moderate to vigorous physical activity, with males (19%) being more active than females (11%). This pattern is similar across other age groups, emphasizing the need for targeted interventions to enhance youth physical activity and health [18].

Physical activity was assessed using subjective methodologies, such as self-reported questionnaires, diaries/logs, interviews [19,20], and direct observation (an observer records an individual’s PA, commonly used in controlled settings, e.g., classrooms, gyms), and objective methodologies, such as device-based monitoring, including motion sensors (pedometers, accelerometers, heart rate monitors), physiological markers, and calorimetry [21,22]. Regarding subjective methodologies, although PA questionnaires are affordable, easy to conduct, and can evaluate PA of a large-scale population, they can present some limitations, including social factors and measurement bias. Children seem to overestimate or underestimate the overall PA level due to a lack of understanding the questions and accurately recalling activities [21,23]. The “Godin-Shephard” Leisure-Time Exercise Questionnaire (GSLTPAQ) is a valid, reliable, and commonly used tool for assessing self-reported physical activity in both adults [19,24,25] and adolescents [26], and in its Greek version, it classifies individuals as “active” or “insufficiently active” [6,27,28,29].

Moreover, for device-based methodologies, the use of step-based metrics as a practical way to record, monitor, and establish physical activity goals could improve individuals’ ability to understand and adopt a physically active lifestyle [30]. Even though the methodologies above objectively estimate PA, there are limitations in applying them to a large-scale population as regards time and cost [21,22], and they also exclude specific activities, such as swimming or bicycle activities [31]. There is an inverse relationship effect of daily steps with significant health outcomes [32]. Similarly, self-monitoring walking activity via pedometer in patients encouraged motivation and a sense of support, enabling them to choose their preferred exercise and adjust their activity levels accordingly [33]. Pedometer validity in PA research has been systematically examined [34], along with comparisons of different manufacturers [34,35,36,37,38]. Regarding, the Yamax Power Walker EX 510 pedometer, the reliability in measuring PA was assessed during Greek traditional dance among adults, further supporting the use of pedometers in diverse PA assessments and research settings [39]. Both subjective and objective methodologies have strengths and limitations, with the choice depending on study goals, population characteristics, and resource availability [40]. However, a concurrent administration of both methodologies could foster a more inclusive qualitative and quantitative evaluation of PA [41].

Children identified with DCD are at higher risk for obesity-related chronic diseases [38] compared to peers with typical development because they have been found to participate less in physical and leisure activities, presenting a higher body mass index and reduced cardiorespiratory fitness, strength, endurance, flexibility, balance, and coordination, as well as a poor body composition [1,42,43,44,45,46] and lower perceived competence [47].

Moreover, recent research studies have shown that children with DCD in the U.S.A. did not meet the Centers for Disease Control and Prevention (CDC) recommendations for PA, lacking enjoyment from participation in organized sports and physical activities [43], while PA in Greek children and adolescents with DCD positively affected motor skill competence, daily function, and social well-being [48]. A comprehensive justification has been provided through the activity deficit hypothesis and the engagement in a negative cycle of inactivity [49,50,51], as children with DCD may avoid participating in team games and physical activities due to common experiences of low social status, e.g., being overlooked or chosen last for sports teams by their peers, resulting in reduced perceived competence and diminished motor skill improvement due to lack of practice. Poor fitness further limits participation in PA, negatively impacting health, well-being, and quality of life [52]. Thus, childhood motor difficulties due to DCD predicted lower PA participation and increased sedentary time in adulthood, emphasizing that motor difficulties continue from childhood to adolescence and adulthood, affecting health and further stressing the need for early targeted PA interventions [53]. The assessment of PA in children and adolescents is essential for motor development and health, especially in those with DCD, as the evidence demonstrates lower participation in PA, particularly in team sports [54,55]. Because subjective and objective PA assessments both have strengths and limitations [40] and there is not a gold standard for assessing children and adolescents, especially those with motor difficulties, it is suggested to use both types of assessment concurrently for a more inclusive evaluation of PA [41] in order to provide vital information for diagnostics and interventions to enhance PA and thus eliminate health and motor development consequences.

As a result, the present study was an effort to assess weekly PA in adolescents facing motor difficulties, with both subjective and objective methods used, given that physical inactivity in this population worsens motor difficulties and general health. Thus, it was anticipated that the prevalence of motor difficulties identified using Movement ABC-2 [9] was within general estimations. Secondly, it was hypothesized that all the study variables would be correlated, and weekly self-reported PA (subjective method) would be associated with pedometer variables (objective method). Finally, weekly self-reported PA and pedometer variables would be differentiated between children identified with motor difficulties and peers with typical motor development. As a result, the purpose of the present study was twofold: firstly, to identify motor difficulties in adolescents, and secondly, to compare objective and subjective weekly PA between adolescents with and without motor difficulties.

## 2. Materials and Methods

### 2.1. Participants and Study Design

**Figure 1:**
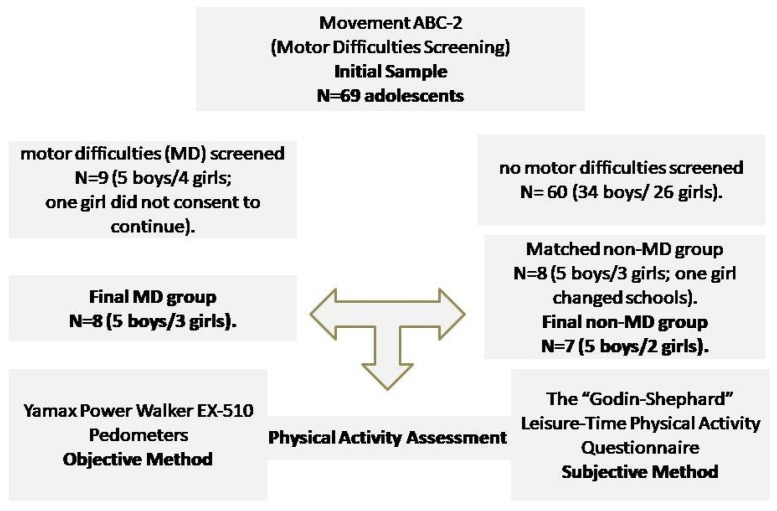
Study design.

The sample was selected by a private secondary school in a small-sized municipality (74,004 residents) of Northern Greece [56]. There are seven (7) public and one (1) private secondary school in this municipality. The private secondary school, with a total of 76 students, was selected to participate in the study as a convenience sample excluding adolescents with intellectual, motor, or sensory disabilities. Moreover, sixty-nine (N = 69) adolescent students agreed to participate in the screening procedure for motor difficulties, completing the Movement ABC-2 [9] motor test. The 69 adolescents were considered representative, as according to statistical calculators, a sample size of at least 64 participants was estimated at the 95% confidence level (standard margin of error of 5%) [57]. According to Movement ABC-2 norms [9], nine (9) adolescents showed motor difficulties with scores below the 15th percentile, and sixty (60) adolescents did not show any difficulties. Afterwards, in order for PA (questionnaires and pedometers) to be assessed, two groups were formed, a motor difficulties group (MD group, N = 9—5 boys and 4 girls) with (1) one girl not consenting to continue, resulting in an MD group with eight participants (N = 8). Moreover, a non-MD group consisted of N= 8 (5 boys and 3 girls), selected voluntary from 60 adolescents without motor difficulties, according to age and gender with the MD group. However, one girl changed schools during the measurements, and the non-MD group finally consisted of 5 boys and 2 girls (N = 7). The study design is depicted in Figure 1.

Regarding participation in extracurricular organized PA, a total percentage of 62.5% (5 children) in the MD group reported participation, while all of the adolescents in the non-MD group reported participation (100%). Moreover, both groups participated in school physical education (PE) lessons twice a week, according to the national PE curriculum. Moreover, the MD and non-MD groups were compared for descriptive purposes according to the demographics of age, body height and body weight, BMI, and weekly frequency of extracurricular organized PA participation using an independent sample *t*-test for equal variances (Levene’s test *p* > 0.05). There were no statistically significant group effects in all the descriptive variables. However, participants in MD group were found to be heavier, and although BMI showed healthy weights for both groups, in the MD group, it tended to be higher. The results for the descriptives are depicted in Table 1.

### 2.2. Materials

#### 2.2.1. The Movement Assessment Battery for Children—Second Edition

The Movement ABC-2 [9] was used for the identification of motor difficulties. It has acceptable validity and reliability, with inter-rater reliability 0.92 to 1.00/test–retest reliability from 0.62 to 0.92 [9], and it includes three age bands. In the present study, the third age band (11–16 years old) was used. It consists of eight items of gross and fine motor skills, grouped into three motor components: manual dexterity, aiming and catching, and balance (static–dynamic). The manual dexterity component includes three items: turning pegs as fast as possible (both hands were tested, measured in number of seconds), triangle with nuts and bolts as fast as possible (a timed bimanual task measured in number of seconds), and a drawing trial task (preferred hand tested, measured in number of errors). One practice attempt was given to each participant, and two formal attempts were recorded. For further calculation, the best (fastest) attempt out of two was measured in turning the pegs and triangle, while the less number of errors was measured in drawing. The aiming and catching component included two items, catching with one hand (both hands were tested) and throwing at a wall target using a tennis ball. Five practice attempts were given to each participant, and ten formal attempts were recorded (the number of successful attempts). The balance component included a static two-board balance task for up to 15 s as a practice attempt and a maximum of two boards up to 30 s as formal attempts (the best attempt with more seconds was recorded). Additionally, there were two dynamic balance tasks: firstly, walking toe-to-heel backwards on a 4.5 m line taped on the floor, with one practice attempt and a maximum of two formal attempts, up to 15 steps, or to the end of the line (the best attempt with the most steps was recorded); and secondly, a zig-zag hopping task, during which each adolescent had one practice attempt and two formal attempts for each leg tested (the best attempt with the most hops to 5 was recorded) [9]. The raw scores were converted into standard scores and percentile equivalents for each motor component and for the total score. The total test scores (up to and including 56) equivalent to a range at or below the 5th percentile denoted significant motor difficulty (red zone); between the 5th to 15th percentiles (total test scores: 57–67 inclusive) suggested the adolescent was “at risk” of having motor difficulty; and if monitoring was required (amber zone) and above the 15th percentile (total test score: any score above 67) denoted that no motor difficulty detected (green zone) [9]. Each adolescent completed the test in about 15–20 min. For the present study, an adolescent with a total score below 67, equivalent to a range at or below the 15th percentile, was considered as having motor difficulty and was assigned to the motor difficulties group (MD group). In addition, any total test score above 67, equivalent to a range above the 16th percentile, was used for the adolescents’ assignment to the non-MD group.

#### 2.2.2. The “Godin–Shephard” Leisure-Time Physical Activity Questionnaire

The “Godin–Shephard” Leisure-Time Physical Activity Questionnaire (GSLTPAQ) was used to assess self-reported PA (subjective method) by reporting how many times the listed activities were completed for more than 15 min in a week, during free time. The validity and reliability of the questionnaire is high [19,20] and it was also reported in its Greek version [28]. According to Godin and Shephard [19], the questionnaire’s score can be computed in two steps. Firstly, the weekly frequencies of strenuous, moderate, and mild activities are multiplied by nine, five, and three, respectively; these three latter values correspond to MET value categories of the activities listed (high scores indicate better physical activity levels). Secondly, the total weekly leisure activity score is computed in arbitrary units by summing the products of the separate components, according to the following formula: Weekly leisure-time activity score = (9 × Strenuous) + (5 × Moderate) + (3 × Mild). However, in this study, the calculation of arbitrary units was also performed according to Godin’s suggestions [20] to use only the reported frequency of strenuous and moderate activities (excluding mild intensity) to compute a health contribution score. In reference to this calculation, the following rule was adopted: 24 units or more: Active (substantial health benefits), 14 to 23 units: Moderately Active (some health benefits), less than 14 units: Insufficiently Active (less substantial or low health benefits) [20].

#### 2.2.3. Yamax Power Walker EX-510 Pedometers (Yamax, Kumamoto, Japan)

Regarding the objective methods for measuring PA, the Yamax Power Walker EX-510 (Yamax, Kumamoto, Japan) pedometers were used. The variables that were evaluated for each individual were the number of steps, distance traveled in kilometers (km), calories in kilocalories (kcal), fat burned in grams (g), and total duration of physical activity (active participation time) in minutes, that is, the time the individuals were active during the day. This type of pedometer enables the recording of movement on three axes. The technical characteristics of the pedometers as given by the manufacturer are as follows: name: Power Walker, Model: EX-510; display: 6-digit dual-series liquid crystals; sensor: 3D accelerometer (steps, calories, fat); measurements: distance, activity time, 24 h clock; size: about 76 × 33.5 × 10 mm; weight: 24 g [58].

### 2.3. Procedures

Informed consent was signed by the adolescents and their parents, who were previously informed about the research requirements and procedures. Participation was voluntary, and the children’s names and any identifying information were not collected. This study was approved by the Institutional Ethics Research Committee. The initial sample of 69 adolescents was screened for motor difficulties using Movement ABC-2. They were classified in the three zones, with significant motor difficulties (red zone), “at risk” of having a motor difficulty, meaning monitoring was required (amber zone), and no motor difficulties detected (green zone) [9]. For the present study, both adolescents in the red zone and amber zone formed the motor difficulties group (MD group) and matched with the non-motor difficulties group (green zone) (non-MD group). The week before the beginning of the pedometer measurement, each adolescent in both groups received a pedometer in a special belt-case for safer recording. They were informed about their use and the importance of placing them on the right hip during recording, as the right pelvic floor is the predominant position of choice for a single recorder [59,60]. In order to eliminate measurement errors based on steps, the stride length was set to 100 cm, adjusting it in centimeters, and the adolescent body weight was set in kilograms, according to the manufacturer’s instructions [39,58]. In addition, the steps of each adolescent were measured walking and running in a line of 20 m using the pedometers and counting steps at the same time as a calibrating procedure [61]. The measurements were started on Monday morning, and the participants used the pedometers every day until Friday, throughout the day. They took them off before going to bed or taking a bath, and also, it was assured that they did not participate in activities such as swimming or biking that would limit the accuracy of the results [31]. Each pedometer variable day score (e.g., number of steps, etc.) was summarized and divided by 5 (the weekdays/Monday to Friday), giving the average score of daily PA. Regarding the PA questionnaires, they were filled in the week after the end of the pedometer measurement as they assessed the previous week’s PA.

### 2.4. Statistical Analysis

For the statistical analysis, SPSS 29.0 was used. Descriptive statistics were performed for better representation of the averages through data tables. Frequency analysis was used in the percentile scores of Movement ABC-2 for the classification of adolescents according to motor difficulties zones. Parametric tests were used, as equal variances were assumed (Levene’s test *p* > 0.05) for all the study variables. An independent samples *t*-test was conducted for descriptive sample variables (age, body weight, body height, BMI, weekly extracurricular organized PA participation) between the two groups. Moreover, Pearson’s correlations were estimated between all the variables. Two one-way multivariate analyses of variance (MANCOVAs) were conducted for the subjective and objective PA variables, examining the main effects for the MD and non-MD groups, following significant correlations. BMI was examined as a covariate along with subjective PA variables, as body height and body weight are decisive factors in determining pedometer outcomes [58] following strong positive statistical correlations. In addition, participation in extracurricular PA weekly frequency was examined as a covariate along with objective PA variables, as the questionnaire used assesses PA in free time and according to strong positive correlations detected [19,20]. To calculate the strength of the results, partial eta-squared were applied (η^2^ = 0.01 small, η^2^ = 0.06 medium, and η^2^ = 0.14 large) [57], and Hedge’s g values were also calculated [62,63]. Statistical significance was set at the *p* < 0.05 level.

## 3. Results

### 3.1. Motor Difficulty Prevalence Using MABC-2

A frequency analysis (N = 69) showed that 7.2% of adolescents appeared to have severe motor difficulties and 5.8% were found to be “at risk” for developing motor difficulties, while 87% did not seem to have any motor difficulties. The results depicted in Table 2 show that a higher percentage (13%) of severe motor difficulties appeared in manual dexterity, while the lowest was found in balance skills (1.4%). Moreover, regarding the gender ratio of severe motor difficulty occurrence, it was found to be 2 boys versus 3 girls, and the ratio for “at risk” was 3 boys versus 1 girl. Thus, the total ratio of motor difficulties was about 1:1, with boys being more susceptible to motor difficulties. Table 3 demonstrates the prevalence of motor difficulties according to gender in all the Movement ABC-2 item scores.

### 3.2. Pearson’s Correlations Among Study Variables

The self-reported weekly PA variables were positively correlated. Moreover, positive correlations emerged between self-reported strenuous PA and total weekly PA. Moreover, positive correlations were found between all the pedometer variables. In addition, BMI was positively correlated with fat burned and calories recorded by the pedometers, while frequency of participation in extracurricular organized PA per week was positively correlated with self-reported strenuous PA and total weekly PA (Table 4).

### 3.3. Differences in the “Godin–Shephard” Leisure-Time Physical Activity Questionnaire Scores Between MD and Non-MD Groups

A frequency analysis regarding the classification of self-reported weekly PA [21] showed that seven (7) adolescents in the MD group out of eight (8) and six (6) out of seven (7) in the non-MD group could be characterized as active, with substantial benefits for their health, indicating total PA for health scores above 24 units [21].

An one-way MANCOVA analysis was estimated to examine the main effects of motor difficulties in the “Godin–Shephard” Leisure-Time Physical Activity Questionnaire variables (subjective measures), using BMI and frequency participation in organized PA per week as covariates. A non-significant MD multivariate effect was revealed (Wilk’s lambda = 0.463, F_(4,8)_ = 2.324, *p* = 0.144, partial eta-squared = 0.537). Follow-up one-way ANCOVAs revealed significant univariate effects for the self-reported weekly frequency of strenuous PA (F_(1,15)_ = 6.757, *p* = 0.025, partial eta-squared = 0.381) and total PA for health (F_(1,15)_ = 10.73, *p* = 0.007, partial eta-squared = 0.494), with the MD group obtaining lower self-reported weekly PA participation. Non-significant univariate effects were found for self-reported weekly moderate PA (F_(1,15)_ = 2.285, *p* = 0.159, partial eta-squared = 0.172) and mild PA (F_(1,15)_ = 1.194, *p* = 0.298, partial eta-squared = 0.098). Regarding the covariates, a multivariate effect was revealed for the self-reported frequency of participation in organized PA per week (Wilk’s lambda = 0.296, F_(4,8)_ = 4.751, *p* = 0.029, partial eta-squared = 0.704). Univariate effects were observed only in the weekly frequency of strenuous PA (F_(1,15)_ = 14.10, *p* = 0.003, partial eta-squared = 0.562) and total weekly PA for health (F_(1,15)_ = 9.73, *p* = 0.010, partial eta-squared = 0.469). On the contrary, BMI did not reveal any significant effect in the self-reported weekly PA (*p* > 0.05). The results are depicted in Table 5.

### 3.4. Differences in Yamax Power Walker EX-510 Pedometer Scores Between MD and Non-MD Groups

An one-way MANCOVA analysis was conducted to examine the main effects of motor difficulties in Yamax Power Walker EX-510 pedometer variables (objective measures), using BMI and frequency participation in organized PA per week as covariates. A significant MD multivariate effect was revealed (Wilk’s lambda = 0.181, F_(5,7)_ = 6.327, *p* = 0.016, partial eta-squared = 0.819). Regarding the covariates, a multivariate effect was revealed for BMI (Wilk’s lambda = 0.119, F_(5,7)_ = 10.406, *p* = 0.004, partial-eta squared = 0.881). Follow-up one-way ANCOVAs revealed non-significant univariate effects for all the pedometer variables (*p* > 0.05).

Hedge’s g effect size (ES) was calculated for each PA variable (objective and subjective) for group comparison (Table 5 and Table 6). Values of 0.15, 0.40, and 0.75 are considered to represent small, medium, and large Ess, respectively [62]. Hedge’s g has been considered approriate in group comparisons with small sample sizes [63]. In terms of motor difficulties’ main effects, there were small to moderate ESs for the subjective PA variables and medium to large ESs for the objective PA variables.

## 4. Discussion

The purpose of the present study was twofold: firstly, to identify motor difficulties in adolescents, and secondly, to compare objective and subjective weekly PA between adolescents with and without motor difficulties.

According to the first research hypothesis regarding the prevalence of motor difficulties in adolescents, the results reveal a slightly high prevalence of severe motor difficulties (7.2%) in the adolescents who participated in the present study, although these results are within general estimations with a range from 2% to 20% [1,3] among the school-aged children population. They are also higher when compared with similar results in Europe, given that in the review by [4], reports from Europe estimated 2%. Moreover, estimates for Asia showed a prevalence of 4% [4], with the exception of a very low percentage in South India, in children and adolescents (0.8%) [5] and 6% in North America, with the exception of a study by [64] in Brazil that estimated a higher prevalence (11.6%) of motor difficulties among public primary school children. However, it was shown that motor difficulties exist in adolescence, and it is crucial to identify these in order to prevent their continuation in adulthood with all the negative consequencies they cause [1,65,66]. These ambiguous findings may be explained by the different assessment tools used (motor assessments, check lists—questionnaires, and selection criteria), sample characteristics (age, gender), cultural variations, and the type of school (public or private), as motor difficulty prevalence estimations are derived mainly from public schools [1,5,64]. The factors above should be taken into account in epidemiological data when interpreting results regarding motor difficulties prevalence [2].

Further analysis regarding the gender ratio of motor difficulty prevalence among adolescents using the Movement ABC-2 provided further insight; the key finding revealed a ratio of 2 boys versus 3 girls, 1:2, presenting significant motor difficulties, while a ratio of 3 boys versus 1 girl (2:1) was presented with “at risk” for having motor difficulties. Regarding the total ratio for both severe and “at risk” for having motor difficulties, it was shown to be 5 boys versus 4 girls, revealing a ratio of about 2:1. These findings are in line with the existing literature, which suggests that motor difficulties affect more boys than girls, in a ratio ranging from 2:1 to 7:1 [1,2], as there may be differences in underlying neurological systems between boys and girls [7]. Moreover, the relationship between motor difficulties and gender is not clear. These gender discrepancies may be due to brain structures, with males having a larger brain size and larger volume but lower density of gray matter than females, whereas females reach peak brain volume earlier and also have differences in cerebral blood flow and thickness of the cortical areas [4,67]. Conversely, recent research has reported varied findings: on one hand, there is a higher prevalence among adolescent boys [6], and on the other hand, there is a higher prevalence among girls, with a boy-to-girl ratio of 1:2, in South India school-aged children (6–15 years old) [5]. This suggests that gender differences in the prevalence of motor difficulties might vary across populations, based on study methods, diagnostic criteria, or cultural factors [2]. These discrepancies emphasize the necessity of standardized assessment methods to accurately determine gender differences in motor difficulty prevalence. Regarding motor domains, the results of the present study show that motor difficulties were present in all three domains, manual dexterity, ball skills, and static and dynamic balance. Moreover, the results show that motor difficulties in balance (static and dynamic) were more prevalent among boys over girls, aligning with other studies suggesting that boys may experience greater postural control challenges [55]. Notably, difficulty with ball skills presented higher gender ratios on behalf of girls versus boys, being in line with the results from the study by Barnett et al. [68], which found that boys were more proficient than girls in object control skills, such as throwing, catching, and kicking.

According to the second research hypothesis, the results indicate significant positive correlations among variablies of self-reported PA (questionnaires); strenuous PA showed a strong positive correlation with total weekly PA for health, indicating that adolescents who engage in strenuous PA reported higher levels of weekly PA for health. Additionally, moderate PA positively correlated with mild PA, demonstrating that adolescents who participated in moderate PA were also more likely to engage in mild PA. These correlations emphasize the consistency of the questionnaire in assessing PA. Furthermore, objective PA variables (pedometers) were all positively correlated, showing a consistency of pedometers in assessing PA. These findings support previous literature in that step count is an important method to assess PA and energy expenditure [16]. In addition, time duration of the activity was strongly correlated with steps, revealing that adolescents who achieved the greatest step count had greater engagement in PA. Regarding body mass index (BMI), there was a significant correlation in calories and fat burned as recorded by the pedometers, suggesting that adolescents with a higher BMI expended more energy during activity. However, BMI was not significantly correlated with self-reported (objective) PA scores; these findings indicate that self-reported PA activity may not adequately convey the impact of BMI on energy expenditure. Additionally, weekly frequency participation in extracuriccular organized PA was significantly correlated with self-reported strenuous PA and total weekly PA for health, highlighting the benefits of sport participation, with emphasis on structured PA, promoting higher PA levels among adolescents, including those with motor difficulties [6,48].

According to the final hypothesis, regarding self-reported weekly PA participation, the results show that adolescents with motor difficulties engaged in significantly lower levels of strenuous PA weekly and total PA for health benefits [20] in comparison with their typically developed peers, providing insights that individuals with motor difficulties are less physically active and avoid participation in PA [46,69]. However, both groups showed adequate self-reported weekly PA participaton with suficient health benefits [20]. As a result, the suggestion that motor difficulties have a negative effect in participation in moderate-to-vigorous (MVPA) and in high-intensity PA [45,55] was not proved for adolescents in the present study. According to the literature, the avoidance of PA participation can result in reduced perceived competence and diminished motor skills due to a lack of practice [49], leading to engagement in a negative cycle of inactivity and the activity deficit hypothesis that potentially lead to adverse effects on PA participation [49,50]. In contrast, there were no differences found in moderate and mild PA, indicating that although adolescents with motor difficulties may struggle with high-intensity exercise, they engage in lower-intensity activities at similar levels to their typically developed peers. This supports the evidence that they probably prefer sedentary or low-intensity activities [55]. Moreover, participation in sports or extracurricular organized PA was found to affect strenuous PA levels and total weekly PA for health benefits. As a result, it seems that sport participation is a crucial variable that should be examined in studies that assess self-reported PA. The fact that adolescents participated slightly less frequent in strenuous PA during a week, but this participation produced sufficient outcomes to their health, could possibly be explained by the type of schoool in which the research was conducted and its curriculum, which included extra-curricular activities, such as sports clubs and drama and art classes, encouraging all students to be more active. These findings reinforce the importance of providing opportunities for adolescents with motor difficulties to develop motor skills in this crucial stage of life, with emphasis on organized PA programs, in a school-based setting [6,48].

Moreover, with regard to the pedometer results, similar results were recorded for both groups. Nonetheless, the results indicate that adolescents in both groups did not meet the criteria recommended for daily step count necessary for health benefits, as mentioned in the international literature [16,17]. Official guidelines argue that adolescents (boys and girls) who achieve between 10,000 and 11,700 steps daily typically reach 60 min MVPA [18], while other research studies suggest that for optimal health, adolescents should aim for at least 10,000 steps per day. In contrast to international recommendations for daily step count, adolescents in the present study failed to reach the necessary step count for health benefits, showing lower average daily step counts. These findings align with previous research indicating that adolescents, particularly those with motor difficulties, tend to have lower PA levels compared to their peers [45,46]. In addition, these results align with evidence of the WHO Regional Office for Europe [18], strengthening the evidence that adolescents aged 13 did not meet the recommended 60 min of daily MVPA. These results emphasize the necessity for targeted interventions to enhance youth PA for health benefits.

Thus, taking into account the results of both objective and subjective PA assessment in the present study, it is obvious that the evidence is challenging. Although the subjective PA assessment (questionnaire) showed sufficient weekly PA for health benefits [20] for both groupsm, with a slight outperformance in typically developed adolescents, the objective PA assessment (pedometers) showed that daily recorded PA in a week, expressed by step counts, did not meet the international recommendations for health benefits [16,17]. In view of our results, in order to provide an inclusive measure of adolescents’ PA, the use of the “Godin–Shephard” Leisure-Time Physical Activity Questionnaire is proposed to receive information about weekly participation in physical activities coupled with pedometers, which provide a more detailed quantification of PA in adolescents both with and without motor difficulties.

### 4.1. Limitations and Strengths

There are some limitations of this study which must be noted. First and foremost, although pedometers provide an objective and device-based assessment of PA, they have some manufacture limitations. Pedometers primarily do not capture the intensity of activity;they only count steps and, furthermore, fail to capture activities such as swimming or cycling, with the possibility of underestimating overall activity levels [40]. Despite this limitation, pedometers remain a valuable tool for assessing PA. A further limitation was the small sample of the present study and the fact that it was derived mainly from a private school, constituting a school-type bias and further affecting the general applicability of the results. However, the type of school used in the present study followed the national school curriculum regarding PE lessons, with optional extracurricular activities at the school facilities (e.g., sport clubs). For mitigating this limitation in future research in public schools, additional questionnaires or daily diaries should be used, coupled with PA assesment tools. However, despite these limitations, the present study provides a holistic approach of PA assessment in adolescents with and without motor difficulties, using both objective and subjective methods. This combination enhances greater reliability and provides a better examination of different variables related to participation in PA, contributing to extending the existing literature, while examining the key factors related to motor difficulties and PA participation.

### 4.2. Future Considerations

Future studies should examine further different socioeconomic backgrounds and residences that may interfere with PA participation and motor difficulties, as the school of the present study was a private school with a high socioeconomic status. In addition, it is recommended that a combination of different device-based instruments coupled with questionnaires should investigate the long-term impacts of interventions based in PA in adolescents with motor difficulties in greater scale.

### 4.3. Practical Implications

The present study provides practical information for physical educators and health professionals who are interested in physical activity and its benefits in physical and motor development and health. The monitoring and motor difficulty identification by movement professionals (physical and occupational therapists), both in school and social contexts, as well as physical education classes—which are mandatory in Greek schools—taught by physical education teachers are critical to promote more varied stimuli and directed motor coordination components required for the adolescent’s motor development. Moreover, given that 60 min of moderate to vigorous physical activity daily provide health benefits [14,15] in children and adolescents and 10,000 to 11,700 steps daily typically help adolescents reach these 60 min of MVPA, the “Godin–Shephard” Leisure-Time Physical Activity Questionnaire coupled with pedometers can provide an affordable, easy, and detailed inclusive assessment of weekly PA in adolescents both with and without motor difficulties in school settings. Our results highlight the necessity of targeted interventions in reducing the health-related negative consequencies of motor difficulties and inactivity.

## 5. Conclusions

To our knowledge, the present study is the first attempt to identify motor difficulties and to assess subjective and objective weekly PA in adolescents with motor difficulties compared to typically developed peers in a private secondary school. The results of this study highlight the prevalence of motor difficulties in adolescents, affecting slightly more boys than girls, and the necessity for an inclusive PA assessment for more robust results, as subjective measures indicated that adolescents were physically active with health benefits, whereas pedometers revealed contradictory results with lower levels of PA in terms of step counts. Taking into account the diverse nature of both measurements, it is suggested that a combination of the “Godin–Shephard” Leisure-Time Physical Activity Questionnaire and pedometers appears to be a promising methodology because it provides a better understanding of the relation between actual and perceived physical activity in children and adolescents. Finally, caution is required when these methods are applied in children and adolescents and especially in those with motor difficulties. All in all, the monitoring and identification of motor difficulties by health professionals and the use of both PA instruments may provide a deeper overview for the elimination of the negative consequences of inactivity and motor difficulties, especially in the first step of the assessment and planning of appropriate PA intervention, in educational and therapeutic settings.

## Figures and Tables

**Table 1:** Means, standard deviations, and effect sizes for group differences in descriptive variables.

	MD Group (N = 8) (M ± SD)	Non-MD Group (N = 7) (M ± SD)	ES g	t_(1,13)_	*p*
Age (years)	12.65 ± 1.04	13.11 ± 0.91	0.46	−0.88	0.39
Body height (m)	1.67 ± 0.12	1.62 ± 0.08	0.48	0.83	0.41
Body weight (kg)	60.68 ± 14.84	49.3 ± 11.49	0.84	1.64	0.12
BMI (kg/m^2^)	21.59 ± 4.05	18.54 ± 3.33	0.81	1.57	0.13
PA participation (times/week)	4.4 ± 1.67	4.3 ± 1.38	0.06	0.13	0.89
PA session duration (h)	1.20 ± 0.44	1.29 ± 0.49	0.19	−0.31	0.76
Stride length (cm)	0.62 ± 0.09	0.62 ± 0.08	0.00	−0.05	0.95

Note: N = 15, M = mean, SD = standard deviation, ES = effect size, MD group = adolescents with motor difficulties, non-MD group = adolescents without motor difficulties, independent samples *t*-test (*t*), statistical significance (*p*) at 0.05. Hedge’s g values of 0.15, 0.40, and 0.75 represent small, medium, and large Ess, respectively.

**Table 2:** Prevalence of motor difficulties in adolescents using Movement ABC-2.

Movement ABC-2 Zones of Motor Difficulty-“Traffic Light System”	Movement ABC-2 (Ν = 69)			
	Total Test Score	Manual Dexterity Score	Aiming and Catching Score	Static/Dynamic Balance Score
	n (%)	n (%)	n (%)	n (%)
No motor difficulties (green zone)	60 (87%)	60 (85.7%)	61 (88.4%)	60 (87%)
“At risk” (amber zone)	4 (5.8%)	0 (0%)	4 (5.8%)	8 (11.6%)
Significant motor difficulties (red zone)	5 (7.2%)	9 (13%)	4 (5.8%)	1 (1.4%)

Note: N = 69, n = the number and % is the respective percentage of adolescents, classified in the three zones of motor difficulties according to Movement ABC-2 scores in manual dexterity, aiming and catching, static/dynamic balance, and total test score (Henderson et al., 2007) [9].

**Table 3:** Prevalence of motor difficulties according to gender using Movement ABC-2.

Movement ABC-2 Zones of Motor Difficulty-“Traffic Light System”	Movement ABC-2 (Ν = 69; 39 Boys and 30 Girls)							
	Total Test Score n (%)		Manual Dexterity Score n (%)		Aiming and Catching Score n (%)		Static/Dynamic Balance Score n (%)	
	Boys	Girls	Boys	Girls	Boys	Girls	Boys	Girls
No motor difficulties (green zone)	34 (87.2%)	26 (86.7%)	34 (87.2%)	26 (86.7%)	61 (88.4%)	24 (80%)	32 (82.1%)	28 (93.3%)
“At risk”(amber zone)	3 (7.7%)	1 (3.3%)	0 (0%)	0(0%)	4 (5.8%)	4 (13.3%)	6 (15.4%)	2 (6.7%)
Significant motor difficulties (red zone)	2 (5.1%)	3 (10%)	5 (12.8%)	4 (13.3%)	4 (5.8%)	2 (6.7%)	1 (2.1%)	0 (0%)

Note: N = 69: 39 boys and 30 girls, n = the number and % is the respective percentage of adolescents, classified in the three zones of motor difficulties according to Movement ABC-2 scores in manual dexterity, aiming and catching, static/dynamic balance, and total test score in boys and girls (Henderson et al., 2007) [9].

**Table 4:** Means, standard deviations, and Pearson’s correlations among study variables.

Variables	M	SD	1	2	3	4	5	6	7	8	9	10	11	12
1. Strenuous PA	3.80	2.07	-											
2. Moderate PA	2.53	1.35	−0.137	-										
3. Mild PA	3.93	2.52	−0.139	0.555 *	-									
4. Total PA health	48.26	20.28	0.944 **	0.031	0.030	-								
5. Number of steps	8445.81	2737.92	0.248	0.144	−0.061	0.204	-							
6. Distancetraveled (km)	5.52	2.26	0.231	0.133	−0.064	0.211	0.930 **	-						
7. Calories (kcal)	335.46	155.09	0.172	0.155	−0.004	0.131	0.878 **	0.953 **	-					
8. Fat burn(g)	46.68	21.39	0.170	0.153	−0.012	0.128	0.883 **	0.953 **	1.00 **	-				
9. Time (min)	78.88	25.69	0.272	0.115	−0.099	0.221	0.988 **	0.951 **	0.904 **	0.908 **	-			
10. Motor difficulties	_	_	0.160	−0.381	−0.194	0.130	−0.210	−0.254	−0.392	−0.401	−0.258	-		
11. BMI	20.17	3.92	−0.048	0.039	0.071	−0.156	0.328	0.338	0.583 *	0.582 *	0.336	−0.401	-	
12. PA participation (times/week)	4.33	1.43	0.780 **	−0.365	−0.326	0.576 *	0.337	0.545	0.492	0.487	0.456	−0.041	0.333	-

Note. N = 15. * *p* < 0.05; ** *p* < 0.01. Motor difficulties (MD) has been coded as 1 (MD adolescent) and 2 (non-MD adolescent).

**Table 5:** Means, standard deviations, effect sizes, and one-way MANCOVAs in self-reported weekly PA for MD and non-MD groups.

Self-Reported Weekly PA	MD (N = 8) (M ± SD)	Non-MD (N = 7) (M ± SD)	ES g	F	*p*	η^2^
Strenuous PA	3.5 ± 2.61	4.16 ± 1.34	0.31	6.75	0.025 *	0.381
Moderate PA	3.0 ± 0.75	2.0 ± 1.73	0.77	2.28	0.159	0.172
Mild PA	4.37 ± 2.87	3.42 ± 2.14	0.37	1.19	0.298	0.098
Total PA Health	45.87 ± 24.85	51.0 ± 14.94	0.24	10.73	0.007 *	0.494

Note: M = mean, SD = standard deviation, ES = effect size, MD group = adolescents with motor difficulties, non-MD group = adolescents without motor difficulties. Statistically significant findings (*, *p* < 0.05). An asterisk (*) denotes a statistical significance between groups. Hedge’s g values of 0.15, 0.40, and 0.75 represent small, medium, and large ESs, respectively.

**Table 6:** Means, standard deviations, effect sizes (ES),and one-way MANCOVAs in pedometer variables for MD and non-MD groups.

Pedometer Weekly Variables	MD (N = 8) (M ± SD)	Non-MD (N = 7) (M ± SD)	ES g	F	*p*	η^2^
Number of steps	8966.4 ± 2848.8	7850.8 ± 2692.2	0.40	1.72	0.216	0.135
Distance traveled (km)	6.04 ± 2.52	4.93 ± 1.95	0.48	0.545	0.476	0.047
Calories (kcal)	390.44 ± 159.7	272.6 ± 133.07	0.79	0.027	0.873	0.002
Fat burned (gr)	54.43 ± 21.80	37.81 ± 18.48	0.81	0.015	0.904	0.001
Time (min)	84.87 ± 26.54	72.02 ± 24.80	0.49	1.78	0.209	0.139

Note: M = mean, SD = standard deviation, ES = effect size, MD group = adolescents with motor difficulties, non-MD group = adolescents without motor difficulties. Hedge’s g values of 0.15, 0.40, and 0.75 represent small, medium, and large ESs, respectively.

## Data Availability

The data presented in this study are available on request from the corresponding author due to privacy reasons.

## Abbreviations

The following abbreviations are used in this manuscript:

DCD	Developmental coordination disorder
PA	Physical activity
PE	Physical education
MVPA	Moderate to vigorous physical activity
GSLTPAQ	Godin–Shephard Leisure Time Physical Activity Questionnaire
MABC-2	Movement Assessment Battery for Children-2
WHO	World Health Organization
CDC	Center for Disease Control
